# P-1961. Quantitative Pneumocystis jirovecii Testing for Prediction of Pneumocystis Disease

**DOI:** 10.1093/ofid/ofaf695.2128

**Published:** 2026-01-11

**Authors:** Revanth S Yendamuri, Lalithaa Thirunavukarasu Murugan, Kyle D Brizendine, Zachary Yetmar, Kelly Marigney, Kamran Kadkhoda, Anisha Misra, Hannah Wang

**Affiliations:** Cleveland Clinic Foundation, Stow, OH; Cleveland Clinic, Cleveland, Ohio; Cleveland Clinic Foundation, Stow, OH; Cleveland Clinic, Cleveland, Ohio; Cleveland Clinic, Cleveland, Ohio; Cleveland Clinic Foundation, Stow, OH; Cleveland Clinic Foundation, Stow, OH; Cleveland Clinic, Cleveland, Ohio

## Abstract

**Background:**

*Pneumocystis jirovecii* pneumonia (PCP) is a serious opportunistic fungal infection. Polymerase chain reaction (PCR) testing of respiratory specimens for *P. jirovecii* is widely recognized as a key diagnostic tool for PCP^1^. However, many individuals can be colonized by *P. jirovecii* without progressing to PCP^2^. This study aims to evaluate the performance characteristics and establish the optimal quantitative threshold of a *P. jirovecii* real-time PCR assay to differentiate proven/probable PCP from colonization.

**Figure 1:**
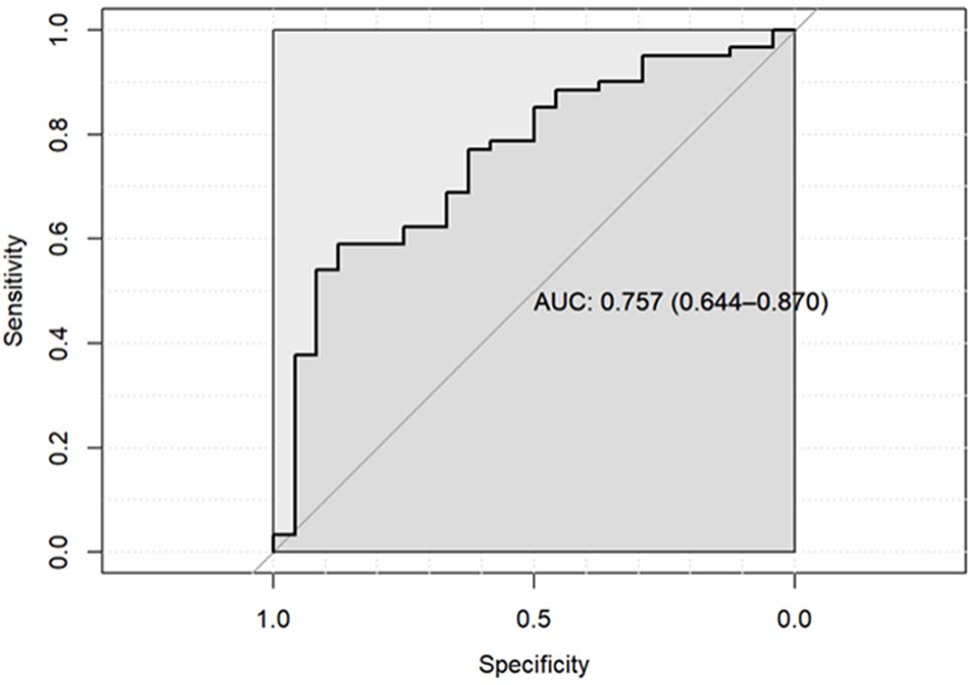
Pneumocystis Threshold Receiver Operating Characteristic Curve

**Table 1:**
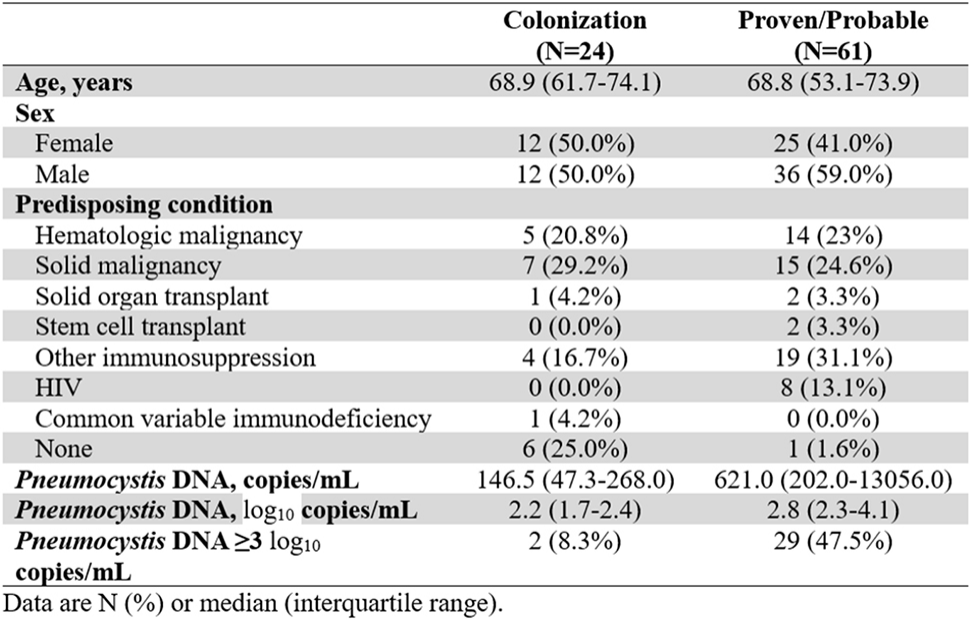
Cohort characteristics

**Methods:**

This single-center cohort study included patients with a positive *P. jirovecii* PCR between September 15^th^, 2023 and September 6^th^, 2024. Patients were categorized as having *P. jirovecii* colonization or proven/probable disease using the EORTC/MSGERC criteria by an infectious disease fellow blinded to the quantitative result. PCR testing was performed on lower respiratory specimens with an assay targeting the multicopy mitochondrial large subunit rRNA (mtLSU) gene. The cutoff of 3-log_10_ (1000 genome copies/mL) was chosen based on a receiver-operating curve conducted on a separate validation cohort^3^.

**Results:**

85 patients were included (24 colonization; 61 proven/probable). Most had at least one predisposing condition for PCP (Table 1). Median *Pneumocystis* DNA was lower in the colonization group vs. the proven/probable group (146.5 copies/mL vs 621.0 copies/mL, p< 0.001). Among PCR-positive individuals, a 3-log_10_ cutoff had a sensitivity of 48% (95% CI 35-61%), specificity 92% (95% CI 73-99%), positive predictive value 94% (95% CI 0.79-0.99%), and negative predictive value 41% (95% CI 28-55%), for prediction of proven/probable disease over colonization (Figure 1).

**Conclusion:**

Among an immunocompromised population, higher quantitative *Pneumocystis* burden in lower respiratory samples was associated with proven/probable disease. However, many patients meeting the criteria for disease had Pneumocystis DNA quantity below the 3-log_10_ threshold. Future studies to establish a separate upper threshold—above which patients meet criteria for disease—and lower threshold—below which patients meet criteria for colonization—may be needed.

**Disclosures:**

Kyle D. Brizendine, MD, Pfizer: Advisor/Consultant Hannah Wang, MD, Cepheid: Grant/Research Support|Hologic: Advisor/Consultant|Hologic: Grant/Research Support|Moderna: Honoraria

